# High-efficient production and biophysical characterisation of nicastrin and its interaction with APPC100

**DOI:** 10.1038/srep44297

**Published:** 2017-03-09

**Authors:** Kun Yu, Ge Yang, Jörg Labahn

**Affiliations:** 1 Centre for Structural Systems Biology (CSSB), DESY, Hamburg, Germany; 2 Institute of Complex Systems, Structural Biochemistry (ICS-6), Forschungszentrum Jülich, Jülich, Germany; 3 Institut für Physikalische Biologie, Heinrich-Heine-Universität Düsseldorf, Germany

## Abstract

Nicastrin, the largest member among the four components of the γ-secretase complex, has been identified to be the substrate recognizer for the proteolytic activity of the complex. Here we report that full-length human nicastrin (hNCT) can be obtained by heterologous expression in *E. coli*. Milligram quantities of the target protein are purified in a two-step purification protocol using affinity chromatography followed by SEC. The FOS-choline 14 purified tetrameric hNCT exhibits a proper folding with 31% α-helix and 23% β-sheet content. Thermal stability studies reveal stable secondary and tertiary structure of the detergent purified hNCT. A physical interaction between nicastrin and the γ-secretase substrate APPC100 confirmed the functionality of hNCT as a substrate recognizer.

The γ-secretase is a unique multi-subunit integral membrane protease which cleaves single path transmembrane proteins inside the membrane. Besides the generation of the apoptotic intracellular peptides from amyloid precursor protein (APP), γ-secretase also plays important roles in the processing of over 90 reported type I transmembrane protein substrates^1^. Four major components are well-known to be responsible for the γ-secretase activity: Presenilin, the catalytic core protein; PEN-2, a potential promotor of presenilin endoproteolysis and a stabilizer of active presenilin fragments; APH-1, a scaffolding protein for the whole γ-secretase complex; and nicastrin, the substrate recognizer.

Nicastrin has the largest molecular weight among the four components of the γ-secretase complex. It consists of 709 amino acids including an N-terminal signal peptide, a large extracellular domain (ECD, residues 34–663) and a single transmembrane (TM) domain followed by a partially membrane-associated C-terminus^2^,^3^. In the γ-secretase complex, nicastrin interacts directly with APH-1 through the TM, while an α-helix and its surrounding loops in the ECD of nicastrin interact with the C-terminus of PEN-2^4^,^5^. Mature nicastrin contains 16 potential N-glycosylation sites which add 30 to 70 kDa to its molecular weight^6^. This extensive glycosylation protects the protein from degradation, but has been found to be not essential for the γ-secretase activity^7^,^8^,^9^,^10^. Therefore, by expression in *E. coli* appears to be a sensible route for this protein to achieve high protein yields.

In addition to the Alzheimer’s disease-related functions, nicastrin has been reported to be relevant for breast cancer^11^,^12^ and acne inversa^13^,^14^. However, the role of nicastrin in the γ-secretase complex is controversial. Initially, the large extracellular domain of nicastrin has been characterized as the substrate recognition executor by binding to the free amino terminus from the ectodomain of γ-secretase substrates (e.g. APP C83 or C99)^2^,^15^,^16^. Particularly, the Glu^333^ of nicastrin and its nearby residues have been proven to be important for substrate recognition^15^,^17^. Antibodies generated against the ECD of nicastrin have shown the capability of γ-secretase inhibition^18^,^19^. Based on the crystal structure of the ECD from a *Dictyostelium purpureum* homolog (sharing 23% identity and 40% similarity with the human nicastrin) and the cryo-EM structure of γ-secretase, a working model of nicastrin as a substrate receptor involving a rotation of a lid region in the ECD has been suggested^5^,^20^. Other studies have pointed out that instead of contributing to the enzymatic function of γ-secretase, nicastrin is involved in the complex maturation (stabilisation)^9^,^21^. And in contrast to earlier results, the free amino group at the N-terminus of the substrate notch seems to be of no importance for nicastrin-induced recognition^22^. Furthermore, deletion of the lid domain in the ECD of nicastrin which has been supposed important for substrate binding does not show the expected regulation effects on the γ-secretase substrate processing^23^. Therefore, a novel steric block mechanism has been proposed which requires nicastrin as a gatekeeper but not for active substrates recruitment^22^.

Upon inhibitor/substrate binding remarkable conformational changes of nicastrin ECD have been observed in the γ-secretase complex isolated from mammalian cells^7^,^24^,^25^. But the basic mechanism of the nicastrin-involved substrate recognition is still unresolved. To further elucidate this topic, here we established an efficient protocol to produce properly folded and functional full-length human nicastrin (hNCT) from the recombinant *E. coli* expression system with a yield of >1 mg protein per liter cell culture, characterised the biophysical properties of the protein and investigated the formation of the initial substrate sub-complex of nicastrin and the M. Alzheimer precursor peptide APPC100, which is processed by the γ-secretase complex to the filament forming Aβ.

## Results

### Construction and expression of hNCT

The first 33 amino acids of nicastrin comprise a cleavable signal peptide and are required for the transport in mammalian cells^26^. In the current study, this signal peptide was not included and the full-length hNCT construct was designed containing residues 34 to 709 and a deca-histidine affinity tag at the N-terminus. Over-production of mammalian membrane proteins using heterologous hosts, especially *E. coli* cells often encounters severe problems such as growth suppression and cell death, which limit the production of the target proteins. For hNCT using a standard BL21 (DE3) *E. coli* cells, better expression was obtained than when using the well-known Walker strain C43, which is often used for membrane proteins^27^. By expression at a temperature of 16 °C, over 50% of BL21 (DE3) expressed hNCT was found in the membrane fraction (see Supplementary Fig. S1).

### Detergent screening and isolation of hNCT

**Figure 1:**
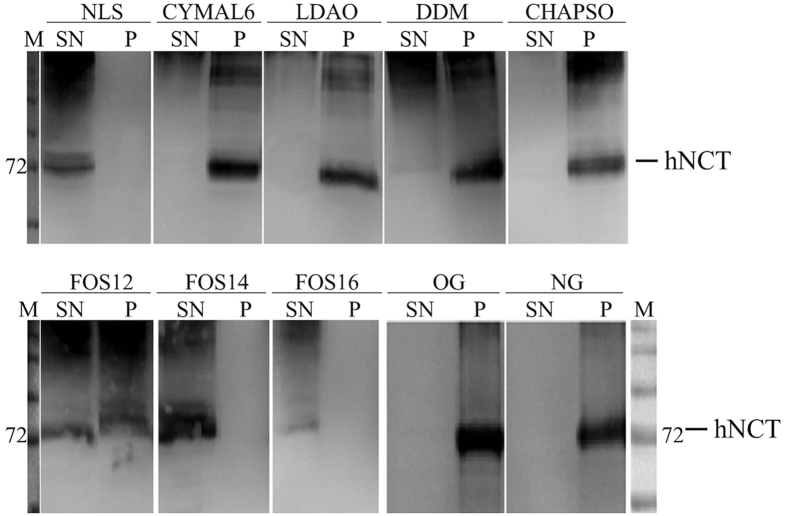
Detergent screening for solubilisation of hNCT. The efficiency of 10 different detergents in solubilising hNCT from *E. coli* membranes was compared. The supernatant (SN) and pellet (P) fractions after solubilising equivalent membrane aliquots were subjected to anti-His tag western blot analysis for hNCT detection (72-kDa marker labelled). Detergents were used at 1% concentration (w/v), except the following: 2% n-Nonyl-β-D-glucopyranoside (NG), n-Octyl-β-D-glucopyranoside (OG) and CHAPSO. As a positive control, N-Lauroylsarcosine sodium salt (NLS) was used to show complete solubilisation of hNCT. LDAO: N, N-Dimethyl-dodecylamine-N- oxide; DDM: n-Dodecyl-β-D-maltoside; FOS: FOS-choline detergents.

Detergents from different classes (non-ionic, zwitterionic and ionic detergents) were screened for solubilisation of hNCT. For most detergents tested, hNCT displayed severe aggregation, which indicated insufficient capacities of these detergents to solubilise hNCT or a low stability of hNCT under these conditions (Fig. 1). CHAPSO was extensively used in purification and activity assays for the γ-secretase complex^28^,^29^, but it showed poor solubilisation ability for *E. coli* expressed hNCT in this study. Most proteins remained in the pellet fraction after solubilisation with NG, OG, and CHAPSO. CYMAL-6, LDAO, and DDM showed partial solubilisation of hNCT, but failed in preventing hNCT from aggregation. The phosphocholine detergents (FOS-choline 12, FOS-choline 14 and FOS-choline 16) exhibited the best solubilisation efficiency. An almost complete solubilisation was achieved by FOS-choline 14 (FOS14) and FOS-choline 16. Nonetheless, hNCT solubilised by FOS-choline16 formed SDS-resistant aggregates. Thus, FOS14 was selected for its solubilisation capacity.

Isolation of hNCT was carried out by a Ni-NTA affinity chromatography and subsequent size exclusion chromatography (SEC). The apparent molecular weight of the FOS14-hNCT complex (peak II, Supplementary Fig. S2) was determined as 362 kDa indicating a tetrameric hNCT. Mass spectrometric (LC-MS/MS) analysis combined with western blotting confirmed that the 72 kDa band represented the full-length hNCT construct including the affinity tag with a sequence coverage of 58% and a score of 480.36 (Sequest HT). The sequence coverage included the complete C-terminus after the transmembrane domain (see Supplementary Fig. S3).

### Biophysical characterization of FOS14-purified hNCT

**Figure 2:**
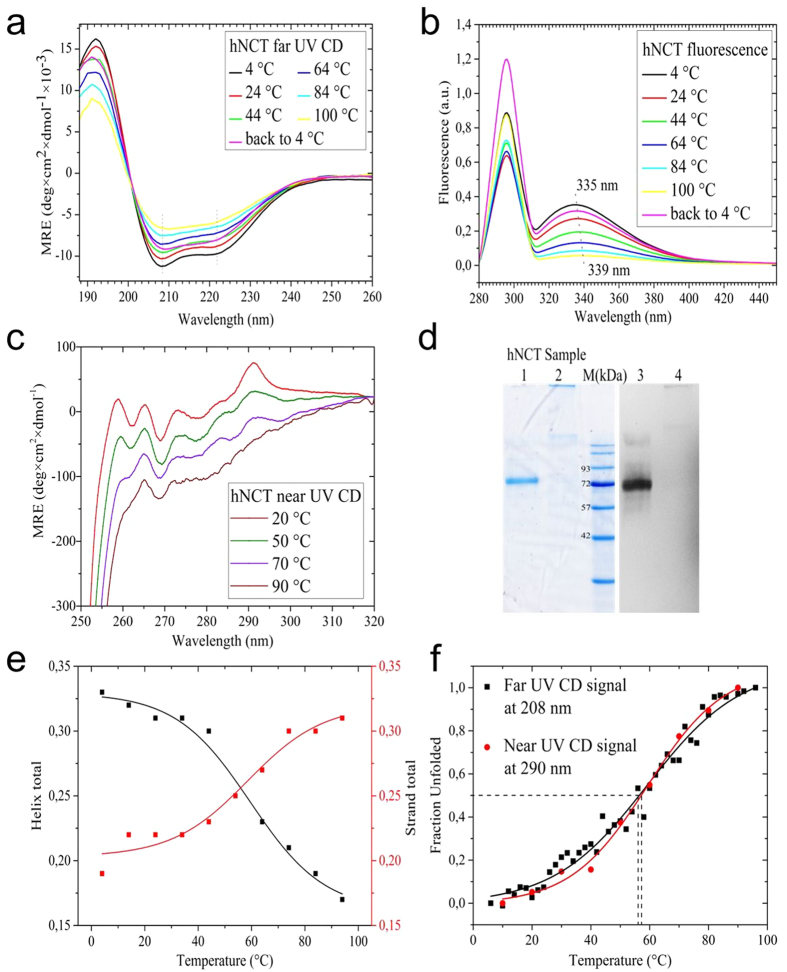
Stability and secondary and tertiary structure of hNCT. (**a**) Far-UV circular dichroism and (**b**) fluorescence spectra of FOS14 purified tetrameric hNCT. For clarity, spectra are shown only for every 20 °C. An additional spectrum was taken after the samples were cooled back to 4 °C (purple). The minima of CD spectra (208 nm and 222 nm) and the maxima of fluorescence are marked with dash lines. (**c**) Tertiary structure assessment by near-UV CD spectra at 20 °C, 50 °C, 70 °C and 90 °C. (**d**) Blue-silver stained SDS-PAGE and western blot of the hNCT sample applied before (Lane 1 and 3) and after (Lane 2 and 4) the thermal stability measurement. (**e**) Thermal transition of secondary structure elements (helix to strand) calculated from deconvolution of far-UV CD-spectra. (**f**) Temperature-induced protein unfolding (based on the far-UV CD signals at 208 nm for secondary structure and near UV CD signal at 290 nm for tertiary structure).

FOS14-purified hNCT samples were analyzed by circular dichroism (CD) and fluorescence spectroscopy to evaluate the protein quality and integrity (secondary and tertiary structure). The hNCT exhibited the characteristic far-UV CD spectrum of a α-helical protein with CD minima at 208 and 222 nm (Fig. 2a). Deconvolution of the CD spectrum using the CDSSTR algorithm showed a secondary structure of 31% α-helix, 23% β-sheet, and 13% turns. The normalized root mean square difference (NRMSD) of 0.02 indicated a good agreement between the calculated and the experimental data sets. The calculated secondary structure of hNCT is broadly in agreement with the secondary structure obtained from the EM structure^5^.

Far-UV CD, tryptophan fluorescence and near-UV CD spectra of hNCT were recorded to evaluate the thermal stability of this detergent-protein complex. A severe loss of helical structure was observed at temperatures above 64 °C in the far-UV CD spectra. The fluorescence spectra of native hNCT showed an emission maximum at 335 nm indicating most of the Trp residues are in a relatively hydrophobic microenvironment^30^. A 4 nm red shift (from 335 nm to 339 nm) of the emission maximum was observed upon thermal denaturation suggesting the presence of heating-induced changes of tryptophan environment from hydrophobic to solvent-exposed (Fig. 2b). The presence of tertiary structure was further confirmed by near-UV CD spectra (Fig. 2c). Significant changes in the emission spectra of the phenylalanine (255 nm–275 nm) and of tryptophan (290 nm–300 nm) were induced by heating. A residual near-UV CD-signal at 100 °C indicated an interaction of the chromophores by aggregation. The aggregation during the denaturation process of hNCT was also observed from the amount of SDS-resistant aggregates (Fig. 2d). A temperature-induced transition of α-helix to β-sheet was observed (Fig. 2e). The CD signal obtained at 208 nm showed a melting temperature (T_m_) of 59.1 ± 1.2 °C for a helical structure (Fig. 2f). The tertiary structure of the hNCT-detergent complex exhibited a thermal stability with a T_m_ of 59.2 ± 2.1 °C (Fig. 2f). These results suggested that the FOS14 purified hNCT represents a well-folded, thermostable protein-detergent complex.

### Interaction of FOS14-purified hNCT and APPC100

In the γ-secretase complex, nicastrin has been proposed to be the substrate recognizer^15^,^31^,^32^. APPC100, a well-known γ-secretase substrate which consists of the C-terminal 100 amino acids of the amyloid precursor protein was employed to investigate the substrate interaction. To determine whether detergent purified hNCT physically associates with APPC100, the N-terminal His-tagged hNCT and the C-terminal Flag-tagged APPC100 were subjected to pull-down assays using Flag resin or Ni-NTA agarose.

**Figure 3:**
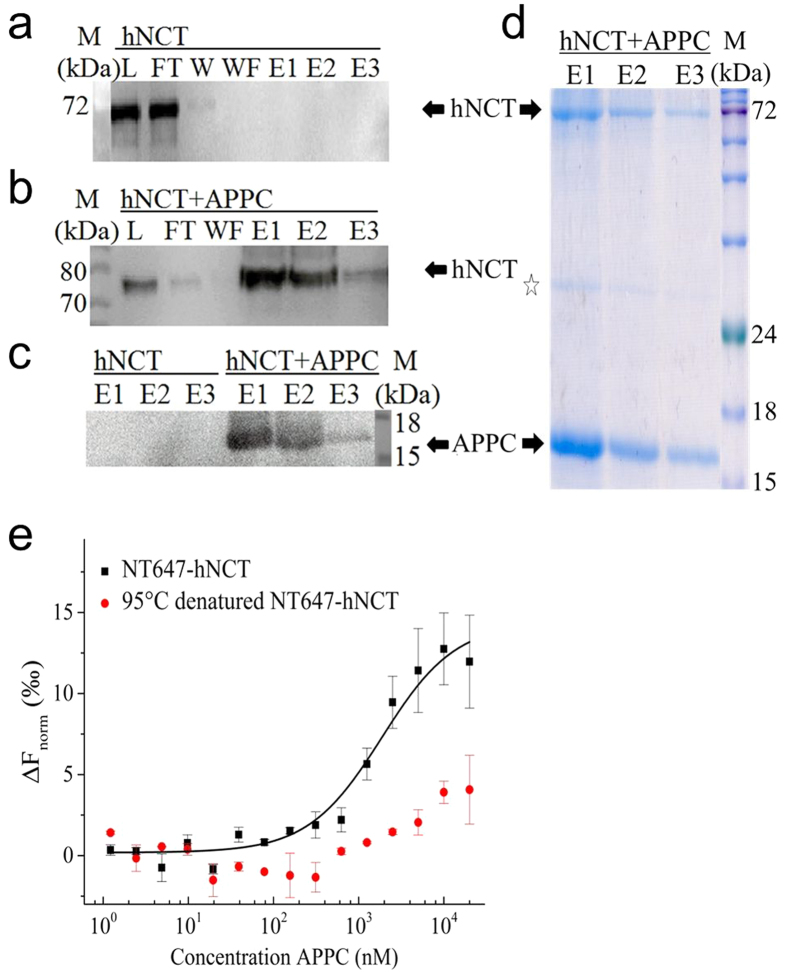
Interaction between hNCT and APPC100. Pull-down assays of hNCT and APPC100 using Flag resin (**a**–**c**) and Ni-NTA agarose (**d**). Anti-His western blot of FOS14 purified hNCT incubated with empty Flag resin (**a**) or APPC100 pre-coated Flag resin (**b**). (**c**) Anti-Flag western blot of APPC100. (**d**) Blue-silver staining of the elution fractions from Ni-NTA agarose (15% SDS-PAGE). L and FT indicated loaded sample and flow-through. W and WF indicated washing steps with samples taken at first and final washing step. E1 to E3 indicated three elution fractions. The presence of hNCT and APPC100 is indicated by a black arrow; black open star indicates APPC100 dimer. (e) MST data for the binding of detergent purified hNCT to APPC100 (native hNCT in black, 95 °C denatured hNCT in red). Data were plotted as normalized signal change as a function of ligand-protein concentration. The error bars represent the s.d. of each data point calculated from three independent thermophoresis measurements.

The FOS14 purified hNCT was captured by APPC100 bound to the Flag resin and co-eluted with APPC100 (Fig. 3b,c), but not in the control experiment in the absence of APPC100 (Fig. 3a). Similarly, Ni-NTA agarose pulled down His-tagged hNCT together with APPC100 from the mixture of separately expressed and detergent purified proteins (Fig. 3d), as well as from a mixture of hNCT and APPC100 which were co-expressed from a cell-free system in the presence of Brij 35 (see Supplementary Fig. S4). These observed interactions were not mediated by unspecific binding of the hydrophobic regions of these two membrane proteins, as there was no retention observed for a control membrane protein by APPC100 on the Flag resin (see Supplementary Fig. S5).

A more detailed assessment of the interaction was obtained using microscale thermophoresis (MST). In the measurements of APPC100 and dye-labelled hNCT, microscale thermophoresis data for concentrations of APPC100 up to 20 μM gave an apparent dissociation constant (KD) in the range of 1–2 μM (Fig. 3e). In contrast, the reverse titration of hNCT to NT647-APPC100 (40 nM) yielded a hill shape MST signal indicated a biphasic event (see Supplementary Fig. S6). The isolation of the hNCT-APPC100 complex by affinity pull-down assays with an apparent dissociation constant of 1–2 μM confirms that detergent purified hNCT binds the γ-secretase substrate APPC100.

## Discussion

Nicastrin consists of 709 amino acids but contains only one transmembrane domain (residue 665 to 697) near the C-terminus of the protein. Nevertheless, this large protein was found in the membrane fraction after cells lysis, though expression in C43 *E. coli* host cells did not improve the yield. Purification and characterization of the *E. coli* expressed full-length hNCT were only successful for protein solubilized by FOS14 from the membrane fraction. Other detergents, such as CHAPSO or DDM, led to either poor solubility or poor homogeneity and purity of the target protein. Previous studies have proven the compatibility of FOS-cholines with γ-secretase purification^33^,^34^. FOS-choline detergents have also been applied successfully for GPCRs^35^,^36^,^37^ and ABC transporters^38^,^39^. The preference of hNCT for the FOS-choline detergents is likely due to the lipid-mimicking properties of these detergents, which assist in retaining a proper folding of the transmembrane domain of hNCT or its relation to the ECD. A binding of phospholipid to the nicastrin TM region has been observed in the cryo-EM structure of γ-secretase^5^, which further supports our observation of detergent preference based on the lipid-mimicking property.

Expression in *E. coli* and solubilisation with FOS 14 allowed isolating the well-defined tetramer of hNCT. An uncharacterized homo-oligomerization of nicastrin has been observed in mammalian cell lines which stably expressed all components of γ-secretase^40^. Thus, the hNCT tetramer can form through at least two possible interaction surfaces: a TM interaction surface which is covered by APH-1 in the γ-secretase complex^5^; and the uncharacterized surface which interacts with other nicastrin^40^.

The tetrameric hNCT exhibited a proper, thermally stable fold with a melting temperature of ca. 59 °C. Upon thermal denaturation, the tetrameric hNCT undergoes an aggregation-dominated process which affects both the near-UV CD and fluorescence measurements. The intrinsic tryptophan fluorescence of hNCT did not show a two-state transition upon thermal denaturation, which was similar to the aggregation affected denaturation of β-galactosidase^41^. In a denaturation accompanied by an aggregation process, aromatic residues may not change their microenvironments substantially, which diminishes the typically observed change of the signal in the near UV CD or fluorescence spectra.

**Figure 4:**
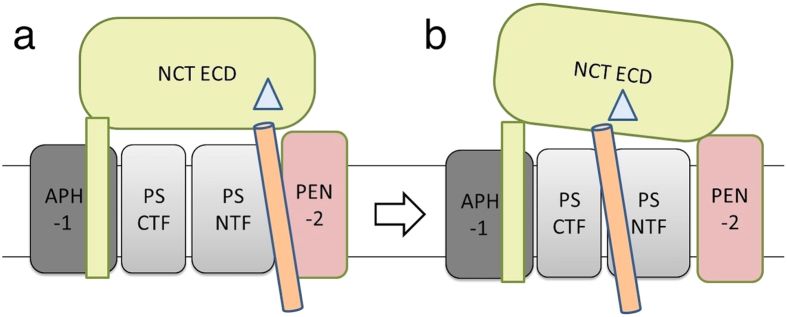
Model of NCT-mediated substrate-recognition and substrate translocation. In γ-secretase, NCT may involve in the formation of an initial substrate docking site (**a**) and the cleavage site (**b**), where the translocated substrate is processed by the catalytic subunit Presenilin. All subunits of the γ-secretase complex are illustrated in the cartoon according to their relative position in the cell membrane (simplified to two black lines): APH-1 (dark grey), Nicastrin (green), Presenilin NTF and CTF (grey), and PEN-2 (pink). The γ-secretase substrate is shoen in orange. The potential interaction site between Nicastrin and the substrate in the ECD of NCT is presented in triangle (light blue).

The exact role of nicastrin in the γ-secretase complex has been controversial. Whether it conducts a residue-based selection and active recruitment of the substrate^15^ or it acts just as a steric block to keep the substrates with larger ectodomain away from the enzyme^22^ is still unclear. In our pull-down assays, the physical interaction of detergent purified hNCT and APPC100 were shown. In the control experiment, another membrane protein did not bind to APPC100 suggesting this interaction was not caused by the random transmembrane domain aggregation but by specific binding. The biphasic binding behaviour of hNCT and labelled APPC100 appears similar to the results reported for the interaction of AMA1 and RON2^42^, which are likely caused by the existence of different forms of sub-complexes. For γ-secretase different sub-complexes, respectively conformations thereof, are discussed^43^ regarding the translocation of the substrates from the initial docking site (Fig. 4a) to the cleavage site (Fig. 4b). Furthermore, detergent purified APPC100 forms oligomers^44^,^45^, which may reduce the apparent affinity and cause more complex binding behaviour when increasing the concentration of hNCT.

The apparent dissociation constants of 1–2 μM between APPC100 and labelled hNCT obtained from MST experiments is considerably larger than the mid-nM range K_D_ estimated for the γ-secretase complex expressed in mammalian cells for the substrate Notch^22^. But the low-μM K_D_ value obtained here is consistent with the initial-docking-site-model for the recognition of substrate by γ-secretase, especially if additional interactions between substrate and PEN-2^43^ in the complex are considered.

The expression of full-length nicastrin in *E. coli* and the solubilisation by FOS14 allowed purification of a stable tetramer of nicastrin that binds the substrate APPC100 to form a sub-complex of the proposed initial-docking-site-complex of γ-secretase.

## Methods

### Plasmid construction and optimized expression of hNCT

The human nicastrin cloning vector was purchased from GeneArt^®^ Gene Synthesis service (gamma-secretase subunit nicastrin, Homo sapiens, Q92542). The synthetic sequence encoded the nicastrin gene with a deca-histidine tag following by a Factor Xa cleavage site at the N-terminus of the protein. It was codon-optimized for the expression in *E. coli* cells. The construct was subcloned into the pQE2 vector (Qiagen) by standard cloning strategies using the 5′-NdeI and 3′-XhoI restriction sites. The construct’s identity was verified by restriction analysis, as well as DNA sequencing (Seqlab, Germany).

The pQE2 plasmid containing hNCT gene was transformed into *E. coli* BL21 (DE3) or C43 (DE3) strains. Cell cultures were grown from single colonies in TB medium supplemented with 50 mg/l Kanamycin at 37 °C to an OD_600_ = 1. The expression of the recombinant protein was induced by addition of 0.4 mM isopropyl-β-thiogalactopyranoside (IPTG). After overnight incubation at 16 °C, cells were harvested by centrifugation (5,000 × g, 30 min, 4 °C). Cells pellet were subsequently subjected to osmotic shock to remove the periplasmic fractions^46^ and frozen at −80 °C for storage.

### Membrane preparation and detergent screen

Cell pellets were stirred for re-suspension in 8 ml lysis buffer (20 mM HEPES, 10% glycerol, pH 7.4) per gram pellet in the cold room for at least 1 hour. EDTA-free protease inhibitor (Roche) 1 tablet/50 ml buffer, 1 mM PMSF, 1 mg/ml Lysozyme and 5 mg/50 g cell pellet DNase I was added to the lysis buffer freshly. The suspension was then passed through an EmulsiFlex-C3 high-pressure homogenizer (Avestin, Inc.) for 5–10 cycles with a pressure of 15 000 to 20 000 psi. Additional 10 mM EDTA and 300 mM NaCl were added to the lysate afterwards. The suspension was centrifuged at 900 × g for 15 min to remove unbroken cells. The obtained supernatant was centrifuged at 10 000 × g for 30 min to remove inclusion body. Finally, membrane fractions were collected by an ultracentrifugation at 100 000 × g for 1 hour. The membranes were washed 3 times with high salt lysis buffer (20 mM HEPES, pH 7.4, 10% Glycerol, 500 mM NaCl), and homogenized by a Dounce homogenizer with storage buffer (20 mM HEPES, pH 7.4, 40% Glycerol, 150 mM NaCl). All centrifugations were performed at 4 °C.

In total, 10 commonly used detergents from all three classes were tested for their solubilisation of hNCT protein. N-Lauroylsarcosine sodium salt (NLS) was used as a positive control for complete solubilisation of the target protein. Solubilisation buffers were prepared to contain 1 or 2% (w/v) detergent. The screening was carried out by diluting membrane stocks into different solubilisation buffers with a ratio of 4–8 ml solubilisation buffer per gram wet cell pellets and incubated overnight at 4 °C with gentle agitation. The mixture was then subjected to ultracentrifugation at 100,000 × g for 1 hour before analysed by SDS-PAGE analysis. The detergent to protein ratio was further optimized based on the expression level: A volume of 6–8 ml solubilisation buffer (0.6% FOS14) per gram wet cell pellet was used to achieve an optimal solubilisation of the target protein.

### Protein purification and western blot detection

Solubilised detergent-protein complexes were incubated with nickel-nitrilotriacetic acid (Ni-NTA) agarose (Qiagen) at 4 °C for 2 hours before transferring the slurry to an empty PD-10 column. In total, 20–30 column volumes (CV) of washing buffer were used in 3 washing steps: 20 × CMC of detergent, 500 mM sodium chloride and imidazole (30 mM). The target protein was eluted in 5 × 1 CV elution buffer with 250 mM imidazole. The eluted fractions were pooled and concentrated using a 50-kDa cut-off concentrator (Amicon^®^ Ultra-15, Merck Millipore Ltd.) to 2 ml before loading to the size exclusion column. A HiLoad Superdex 200 prep grade column was connected to an Akta Explorer FPLC system (GE Healthcare) and equilibrated with SEC buffer (20 mM HEPES, pH 7.4, 10% Glycerol, 150 mM NaCl, 2 CMC detergent). SEC was performed at 0.2 ml/min.

10% SDS-PAGE gels were used for visualizing hNCT (15% SDS-PAGE gels for APPC100) with prestained protein molecular weight marker (PS-105, Jena Bioscience) or BenchMark™ Protein Ladder (10747012, ThermoFisher Scientific). Protein samples were prepared by adding 5 × loading buffer and incubated at 46 °C for 10 min prior to avoid membrane protein aggregation. For western blotting, the transfer was performed using 0.45 mm nitrocellulose membrane and Trans-Blot^®^ Turbo™ Transfer System (Bio-Rad Laboratories, Inc.). After membrane blocking in milk (5% non-fat dried milk in TBST) for 1 hour, membranes were incubated with the respective antibody: a monoclonal anti-polyhistidine antibody (A7058, Sigma) for hNCT detection; or OctA-Probe Antibody (H-5) (sc-166355, Santa Cruz Biotechnology) for APPC100 detection. For visualisation of enhanced chemiluminescence (ECL) the Gel Doc XR^+^ System (Bio-Rad Laboratories, Inc.) was used.

### Secondary structure and thermal stability analysis by CD and fluorescence spectroscopy

Far-UV CD and fluorescence spectra were measured using purified hNCT proteins at concentrations of 0.1–0.2 mg/ml. Spectra were collected with an 111-QS quartz sample cell (Hellma, 1 mm path length). Near-UV CD spectra were measured using 1–1.5 mg/ml detergent purified hNCT in an 1 cm path-length sample cell (Aviv). CD spectra for the secondary structure estimation were recorded on a CD spectrometer (Aviv Associates model 425) from 185 nm to 260 nm at 4 °C with a 1 nm step size and an averaging time of 9 seconds. Difference spectra for purified hNCT were obtained by subtracting the CD buffer blank (10 mM NaH_2_PO_4_, 100 mM NaF and 2 × CMC FOS14). Deconvolutions of obtained CD data were performed using CDSSTR with a reference set SMP180.

Thermal stability of hNCT was measured using the upgraded Aviv 425 circular dichroism spectrometer equipped with fluorescence emission-scanning monochromator which allows simultaneous collection of CD and fluorescence data. Additionally, fluorescence and light scattering were monitored with a fluorescence scan at 90° to the incident beam starting at the excitation wavelength. Temperature scans (from 4 °C to 98 °C) were performed at a heating rate of 1 °C per minute. CD spectra were collected with a bandwidth of 1 nm and an averaging time of 6 seconds. An excitation wavelength of 295 nm was used to selectively excite Trp. The fluorescence emission spectra were collected from 450 nm to 300 nm with a bandwidth of 2 nm and an averaging time of 1 second. The raw data were smoothed and analysed using Origin 8 software.

### Pull-down assays of hNCT and APPC100

The detergent purified hNCT was obtained as described above. APPC100 was purified as described elsewhere^47^,^48^. Protein or protein mixture was incubated at 4 °C for 2 hours with Ni-NTA agarose (Qiagen) or overnight with anti-DYKDDDDK G1 affinity resin (GeneScript^®^). The Ni-NTA resin was washed and the hNCT-APPC100 eluted from Ni-NTA with 250 mM imidazole as described for the hNCT purification. For the protein bound to anti-DYKDDDDK G1 affinity resin, excessive washing buffer (20 mM HEPES, pH 7.4, 150 mM NaCl, 2 × CMC FOS14) was applied to remove the unspecific binding of hNCT. The APPC100-hNCT sub-complexes were eluted from flag resin with 100 mM glycine, pH 2.5, and neutralized with 0.1 volumes of 1 M HEPES, pH 9. The elution fractions were collected in three fractions with 1 × CV each fraction. Samples from the loading and washing steps were normalized for concentration and loaded to the SDS-PAGE. Concentrated elution fractions were analysed using SDS-PAGE by blue-silver staining or immunoblotting.

### MST analyses

MST analysis was performed using NanoTemper Monolith NT.115, as recently described^42^,^49^. Purified hNCT or APPC100 were fluorescence-labeled using the NanoTemper protein labeling kit RED-NHS. The labeling procedure was performed at 4 °C overnight. 20 nM of NT647-labeled hNCT was incubated with different concentrations of APPC100 (from 1 nM to 40 μM) and loaded into Monolith NT Capillaries. In the reverse experiments, 40 nM of NT647-labeled APPC100 was titrated by 0.2 nM to 10 μM of hNCT. Measurements were performed at 20 °C by using 40% LED power and 40% IR-laser power. Thermophoresis analyses were also carried out on 20 or 60% LED power and 40% IR-Laser power for comparison.

## Additional Information

**How to cite this article**: Yu, K. *et al*. High-efficient production and biophysical characterisation of nicastrin and its interaction with APPC100. *Sci. Rep.* **7**, 44297; doi: 10.1038/srep44297 (2017).

**Publisher's note:** Springer Nature remains neutral with regard to jurisdictional claims in published maps and institutional affiliations.

## Supplementary Material

Supplementary Information

## References

[b1] Haapasalo A. & Kovacs D. M. The many substrates of presenilin/γ-secretase. J. Alzheimers. Dis. 25, 3–28 (2011). 21335653 10.3233/JAD-2011-101065 PMC3281584

[b2] Yu G. . Nicastrin modulates presenilin-mediated notch/glp-1 signal transduction and betaAPP processing. Nature 407, 48–54 (2000). 10993067 10.1038/35024009

[b3] Li Y., Liew L. S. Y., Li Q. & Kang C. Structure of the transmembrane domain of human nicastrin-a component of γ-secretase. Sci. Rep. 6, 19522 (2016). 26776682 10.1038/srep19522 PMC4726005

[b4] Morais V. A. . The Transmembrane Domain Region of Nicastrin Mediates Direct Interactions with APH-1 and the γ-Secretase Complex. J. Biol. Chem. 278, 43284–43291 (2003). 12917438 10.1074/jbc.M305685200

[b5] Bai X. . An atomic structure of human γ-secretase. Nature 525, 212–217 (2015). 26280335 10.1038/nature14892 PMC4568306

[b6] Schedin-Weiss S., Winblad B. & Tjernberg L. O. The role of protein glycosylation in Alzheimer disease. FEBS J. 281, 46–62 (2014). 24279329 10.1111/febs.12590

[b7] Shirotani K. . Gamma-secretase activity is associated with a conformational change of nicastrin. J. Biol. Chem. 278, 16474–7 (2003). 12644462 10.1074/jbc.C300095200

[b8] Kimberly W. T. . Complex N-linked Glycosylated Nicastrin Associates with Active γ-Secretase and Undergoes Tight Cellular Regulation. J. Biol. Chem. 277, 35113–35117 (2002). 12130643 10.1074/jbc.M204446200

[b9] Herreman A. . gamma-Secretase activity requires the presenilin-dependent trafficking of nicastrin through the Golgi apparatus but not its complex glycosylation. J. Cell Sci. 116, 1127–1136 (2003). 12584255 10.1242/jcs.00292

[b10] Zhang X. . Identification of a tetratricopeptide repeat-like domain in the nicastrin subunit of -secretase using synthetic antibodies. Proc. Natl. Acad. Sci. 109, 8534–8539 (2012). 22586122 10.1073/pnas.1202691109 PMC3365189

[b11] Lee S. H., Sharma M., Sudhof T. C. & Shen J. Synaptic function of nicastrin in hippocampal neurons. Proc. Natl. Acad. Sci. 111, 8973–8978 (2014). 24889619 10.1073/pnas.1408554111 PMC4066509

[b12] Lombardo Y. . Nicastrin regulates breast cancer stem cell properties and tumor growth *in vitro* and *in vivo*. Proc. Natl. Acad. Sci. 109, 16558–16563 (2012). 23012411 10.1073/pnas.1206268109 PMC3478621

[b13] Wang B. . γ-Secretase Gene Mutations in Familial Acne Inversa. Science (80−) 330, 1065–1065 (2010). 10.1126/science.1196284 20929727

[b14] Zhang S., Meng J., Jiang M. & Zhao J. Characterization of a Novel Mutation in the NCSTN Gene in a Large Chinese Family with Acne Inversa. Acta Derm. Venereol. 96, 408–409 (2016). 26463457 10.2340/00015555-2259

[b15] Shah S. . Nicastrin Functions as a γ-Secretase-Substrate Receptor. Cell 122, 435–447 (2005). 16096062 10.1016/j.cell.2005.05.022

[b16] Esler W. P. . Activity-dependent isolation of the presenilin- gamma-secretase complex reveals nicastrin and a gamma substrate. Proc. Natl. Acad. Sci. 99, 2720–2725 (2002). 11867728 10.1073/pnas.052436599 PMC122414

[b17] Dries D. R. . Glu-333 of Nicastrin Directly Participates in γ-Secretase Activity. J. Biol. Chem. 284, 29714–29724 (2009). 19729449 10.1074/jbc.M109.038737 PMC2785603

[b18] Hayashi I. . Single Chain Variable Fragment against Nicastrin Inhibits the γ-Secretase Activity. J. Biol. Chem. 284, 27838–27847 (2009). 19684016 10.1074/jbc.M109.055061 PMC2788834

[b19] Hayashi I. . Neutralization of the γ-secretase activity by monoclonal antibody against extracellular domain of nicastrin. Oncogene 31, 787–798 (2012). 21725355 10.1038/onc.2011.265 PMC4058788

[b20] Xie T. . Crystal structure of the γ-secretase component nicastrin. Proc. Natl. Acad. Sci. 111, 13349–13354 (2014). 25197054 10.1073/pnas.1414837111 PMC4169925

[b21] Chávez-Gutiérrez L. . Glu332 in the nicastrin ectodomain is essential for γ-secretase complex maturation but not for its activity. J. Biol. Chem. 283, 20096–20105 (2008). 18502756 10.1074/jbc.M803040200

[b22] Bolduc D. M., Montagna D. R., Gu Y., Selkoe D. J. & Wolfe M. S. Nicastrin functions to sterically hinder γ-secretase–substrate interactions driven by substrate transmembrane domain. Proc. Natl. Acad. Sci. 113, E509–E518 (2016). 26699478 10.1073/pnas.1512952113 PMC4747693

[b23] Zhang X. . Evidence That the ‘Lid’ Domain of Nicastrin Is Not Essential for Regulating γ-Secretase Activity. J. Biol. Chem. 291, 6748–6753 (2016). 26887941 10.1074/jbc.C115.701649 PMC4807262

[b24] Li Y. . Structural Interactions between Inhibitor and Substrate Docking Sites Give Insight into Mechanisms of Human PS1 Complexes. Structure 22, 125–135 (2014). 24210759 10.1016/j.str.2013.09.018 PMC3887256

[b25] Elad N. . The dynamic conformational landscape of -secretase. J. Cell Sci. 128, 589–598 (2015). 25501811 10.1242/jcs.164384 PMC4311135

[b26] Hansson C. A. . Nicastrin, Presenilin, APH-1, and PEN-2 Form Active γ-Secretase Complexes in Mitochondria. J. Biol. Chem. 279, 51654–51660 (2004). 15456764 10.1074/jbc.M404500200

[b27] Miroux B. & Walker J. E. Over-production of Proteins inEscherichia coli: Mutant Hosts that Allow Synthesis of some Membrane Proteins and Globular Proteins at High Levels. J. Mol. Biol. 260, 289–298 (1996). 8757792 10.1006/jmbi.1996.0399

[b28] Fraering P. C. . Purification and Characterization of the Human γ-Secretase Complex. Biochemistry 43, 9774–9789 (2004). 15274632 10.1021/bi0494976

[b29] Li Y. . Photoactivated gamma-secretase inhibitors directed to the active site covalently label presenilin 1. Nature 405, 689–94 (2000). 10864326 10.1038/35015085

[b30] Munishkina L. A. & Fink A. L. Fluorescence as a method to reveal structures and membrane-interactions of amyloidogenic proteins. Biochim. Biophys. Acta - Biomembr. 1768, 1862–1885 (2007). 10.1016/j.bbamem.2007.03.015 17493579

[b31] Chávez-Gutiérrez L. . Glu(332) in the Nicastrin ectodomain is essential for gamma-secretase complex maturation but not for its activity. J. Biol. Chem. 283, 20096–105 (2008). 18502756 10.1074/jbc.M803040200

[b32] Bolduc D. M. & Wolfe M. S. Structure of nicastrin unveils secrets of γ-secretase. Proc. Natl. Acad. Sci. 111, 14643–14644 (2014). 25267656 10.1073/pnas.1416637111 PMC4205669

[b33] Zhou S., Zhou H., Walian P. J. & Jap B. K. CD147 is a regulatory subunit of the -secretase complex in Alzheimer’s disease amyloid -peptide production. Proc. Natl. Acad. Sci. 102, 7499–7504 (2005). 15890777 10.1073/pnas.0502768102 PMC1103709

[b34] Zhou H., Zhou S., Walian P. J. & Jap B. K. Dependency of γ-secretase complex activity on the structural integrity of the bilayer. Biochem. Biophys. Res. Commun. 402, 291–296 (2010). 20937251 10.1016/j.bbrc.2010.10.017 PMC2981652

[b35] Ren H. . High-Level Production, Solubilization and Purification of Synthetic Human GPCR Chemokine Receptors CCR5, CCR3, CXCR4 and CX3CR1. PLoS One 4, e4509 (2009). 19223978 10.1371/journal.pone.0004509 PMC2637981

[b36] Cook B. L. . Large-scale production and study of a synthetic G protein-coupled receptor: Human olfactory receptor 17-4. Proc. Natl. Acad. Sci. 106, 11925–11930 (2009). 19581598 10.1073/pnas.0811089106 PMC2715541

[b37] Wang X., Corin K., Rich C. & Zhang S. Study of two G-protein coupled receptor variants of human trace amine-associated receptor 5. Sci. Rep. 1, 102 (2011). 22355620 10.1038/srep00102 PMC3216587

[b38] Galián C. . Optimized Purification of a Heterodimeric ABC Transporter in a Highly Stable Form Amenable to 2-D Crystallization. PLoS One 6, e19677 (2011). 21602923 10.1371/journal.pone.0019677 PMC3094339

[b39] Infed N., Hanekop N., Driessen A. J. M., Smits S. H. J. & Schmitt L. Influence of detergents on the activity of the ABC transporter LmrA. Biochim. Biophys. Acta - Biomembr 1808, 2313–2321 (2011). 10.1016/j.bbamem.2011.05.016 21651889

[b40] Walker E. S., Martinez M., Wang J. & Goate A. Conserved residues in juxtamembrane region of the extracellular domain of nicastrin are essential for gamma-secretase complex formation. J. Neurochem. 98, 300–309 (2006). 16805816 10.1111/j.1471-4159.2006.03881.x

[b41] Kishore D. . Thermal, Chemical and pH Induced Denaturation of a Multimeric β-Galactosidase Reveals Multiple Unfolding Pathways. PLoS One 7, e50380 (2012). 23185611 10.1371/journal.pone.0050380 PMC3503960

[b42] Seidel S. A. I. . Microscale thermophoresis quantifies biomolecular interactions under previously challenging conditions. Methods 59, 301–315 (2013). 23270813 10.1016/j.ymeth.2012.12.005 PMC3644557

[b43] Fukumori A. & Steiner H. Substrate recruitment of γ-secretase and mechanism of clinical presenilin mutations revealed by photoaffinity mapping. EMBO J. (2016). 10.15252/embj.201694151 PMC4883025 27220847

[b44] Beel A. J. . Nonspecificity of Binding of γ-Secretase Modulators to the Amyloid Precursor Protein. Biochemistry 48, 11837–11839 (2009). 19928774 10.1021/bi901839d PMC2794937

[b45] Botev A. . The Amyloid Precursor Protein C-Terminal Fragment C100 Occurs in Monomeric and Dimeric Stable Conformations and Binds γ-Secretase Modulators. Biochemistry 50, 828–835 (2011). 21186781 10.1021/bi1014002

[b46] Magnusdottir A., Johansson I., Dahlgren L.-G., Nordlund P. & Berglund H. Enabling IMAC purification of low abundance recombinant proteins from E. coli lysates. Nat. Methods 6, 477–478 (2009). 19564847 10.1038/nmeth0709-477

[b47] Kimberly W. T. . Notch and the Amyloid Precursor Protein Are Cleaved by Similar γ-Secretase(s). Biochemistry 42, 137–144 (2003). 12515548 10.1021/bi026888g

[b48] Li Y. . Presenilin 1 is linked with gamma -secretase activity in the detergent solubilized state. Proc. Natl. Acad. Sci. 97, 6138–6143 (2000). 10801983 10.1073/pnas.110126897 PMC18571

[b49] Jerabek-Willemsen M. . MicroScale Thermophoresis: Interaction analysis and beyond. J. Mol. Struct. 1077, 101–113 (2014).

