# Supplementary material for: High-efficient production and biophysical characterisation of nicastrin and its interaction with APPC100
Source: Sci Rep. 2017 Mar 9;7:44297. doi: 10.1038/srep44297 (PMC5343570; doi:10.1038/srep44297)
Supplement: Supplementary Information [file srep44297-s1.pdf]

Supplementary Information

High-efficient production and biophysical characterisation of nicastrin and its interaction with APPC100

Kun Yu [a,b,§] , Ge Yang [a,b,§] and Jörg Labahn [a,b,c,*]

[a] Centre for Structural Systems Biology (CSSB), DESY, Hamburg, Germany

[b] Institute of Complex Systems, Structural Biochemistry (ICS-6), Forschungszentrum Jülich, Jülich, Germany

c Institut für Physikalische Biologie, Heinrich-Heine-Universität Düsseldorf, Germany

§ These authors contributed equally to this work.

* Correspondence and requests for materials should be addressed to J.L. (email: j.labahn@fz-juelich.de)

Supplementary Information

Expression of hNCT

Nicastrin, the largest component of the γ-secretase complex, consists of 709 amino acids but only one transmembrane domain (residue 665 to 697) near the C-terminus of the protein. Therefore, the expression of hNCT was investigated using *E.coli* BL21 (DE3) and the well-known Walker strain C43 (DE3) which is often utilized for membrane protein expression. The growth curves for these two strains carrying the hNCT plasmid did not show significant differences indicating that both strains tolerated the expression equally, though comparison with a reference protein indicated moderate growth retardation for the Walker strain (**Supplementary Fig. S1** a).

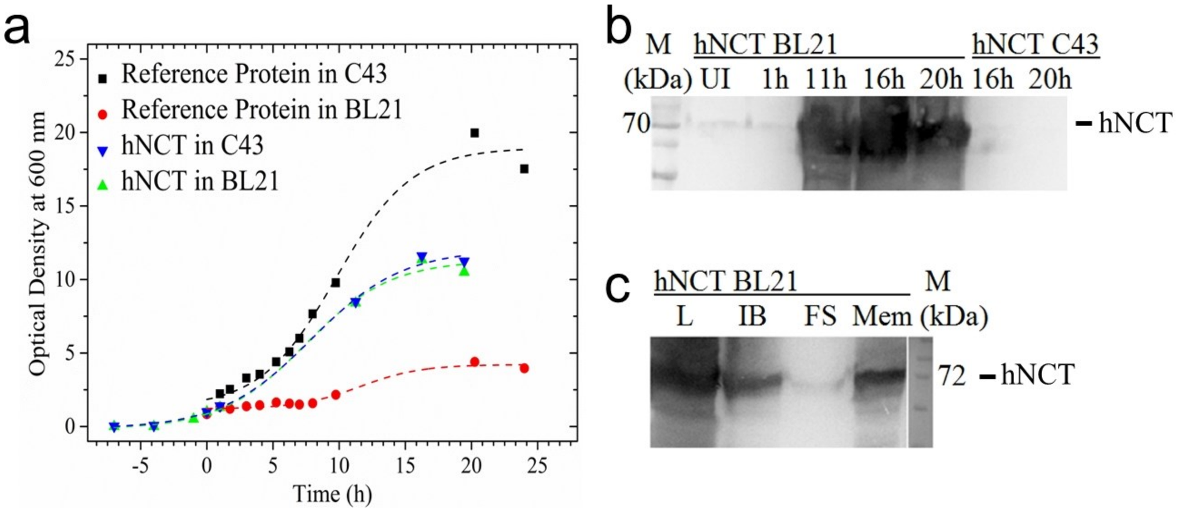

Supplementary Figure S1 Selection of *E.coli* host strains for inducible expression and distribution of hNCT.

(a) The cell growth curves for hNCT and reference membrane protein in *E.coli* C43 and BL21 (DE3) cells. (b) Time-course analysis of hNCT expression. UI represent the un-induced cells. Weak signals of C43 (DE3) expressed hNCT were only detectable after overexposure. (c) Western blot analyse of hNCT distribution. L, cell lysate; IB, inclusion body; FS, final supernatant; Mem, membrane fraction.

Maximal expression of hNCT was reached at 16 hours after induction of the BL21 (DE3) cells (**Supplementary Fig. S1** b). The expression level was much lower in the C43 (DE3) strain. This might be due to the low transcription level of the target gene in this cell strain. Analysing the distribution of hNCT, a considerable amount of the target protein was found in inclusion bodies indicating incorrect folding. Lowering the induction temperature decreased the amount of this fraction. After optimizing the expression conditions, specifically, the induction temperature to 16 °C, over 50% of BL21 (DE3) expressed hNCT was found in the membrane fraction (**Supplementary Fig. S1** c).

Supplementary Information

Purification of recombinant hNCT

The purity of hNCT captured by affinity chromatography could be optimized by alternatively washing the affinity resin with buffers of high detergent and high salt concentration after loading the protein.

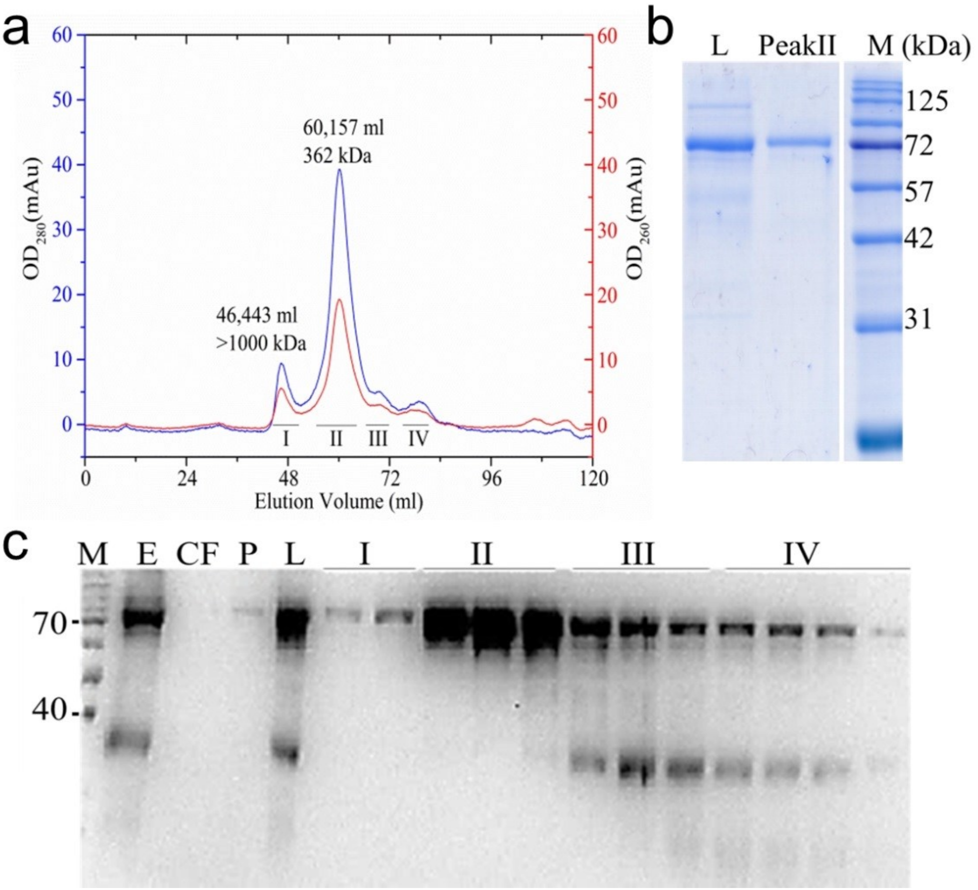

Supplementary Figure S2 Large-scale purification of hNCT.

(a) The purification of hNCT is illustrated as SEC profile from HiLoad Superdex 200 prep grade column. Blue-silver staining SDS-PAGE (b) and western blot (c) of the peak fractions showed a pure hNCT in the peak II. L: hNCT protein eluted from Ni-NTA affinity purification before loading to SEC; E: hNCT protein eluted from Ni-NTA affinity purification; CF: the flow through during the concentration; P: pellet before loading to SEC; I, II, III, IV corresponds to the peak region in the SEC profile.

When applied to SEC, hNCT purified in FOS14 showed four peaks in the SEC profile. They represent oligomers, aggregates of the target protein and degradation fragments, respectively (**Supplementary Fig. S2** a and c). A symmetric and dominating peak (II) containing full-length hNCT was well-separated from impurities or degradation fragments. Based on a calibration curve, the apparent molecular weight of the FOS14-hNCT complex in peak II was determined as 362 kDa indicating a tetrameric hNCT. The pooled fractions of hNCT in the main peak exhibited over 95% purity (**Supplementary Fig. S2** b, Peak II).

Supplementary Information

Primary sequence analysis of isolated hNCT

Although the construct used for hNCT expression has a predicted molecular weight of 77 kDa, it migrated at 72 kDa on the 10% SDS-PAGE. Mass spectrometric (LC-MS/MS) analysis combined with western blotting confirmed that the 72 kDa band represented the full-length hNCT construct including the affinity tag which was confirmed by Western blot.

```
1     MHHHHHHHHHHAIEGRNSVERKIYIPLNKTAPCVRLLNATHQIGCQSSISGDTGVIHVVE
61    KEEDLQWVLTDGPNPPYMVLLESKHFTRDLMEKLKGRTSRIAGLAVSLTKPSPASGFSPS
121   VQCPNDGFGVYSNSYGPEFAHCREIQWNSLGNGLAYEDFSFPIFLLEDENETKVIKQCYQ
181   DHNLSQNGSAPTFPLCAMQLFSHMHAVISTATCMRRSSIQSTFSINPEIVCDPLSDYNVW
241   SMLKPINTTGTLKPDDRVVVAATRLDSRSFFWNVAPGAESAVASFVTQLAAAEALQKAPD
301   VTTLPRNVMFVFFQGETFDYIGSSRMVYDMEKGKFPVQLENVDSFVELGQVALRTSLELW
361   MHTDPVSQKNESVRNQVEDLLATLEKSGAGVPAVILRRPNQSQPLPPSSLQRFLRARNIS
421   GVVLADHSGAFHNKYYQSIYDTAENINVSYPEWLSPEEDLNFVTDTAKALADVATVLGRA
481   LYELAGGTNFSDTVQADPQTVTRLLYGFLIKANNSWFQSILRQDLRSYLGDGPLQHYIAV
541   SSPTNTTYVVQYALANLTGTVVNLTREQCQDPSKVPSENKDLYEYSWVQGPLHSNETDRL
601   PRCVRSTARLARALSPAFELSQWSSTEYSTWTESRWKDIRARIFLIASKELELITLTVGF
621   GILIFSLIVTYCINAKADVLFIAPREPGAVSY
```

Supplementary Figure S3 Peptides identification of hNCT by LC-MS/MS analysis.
Matched peptides detected from MS are shown in shading. Predicted transmembrane region is underlined. Sequence coverage of 58% (Score 480.36, Sequest HT)

Supplementary Information

Co-expression of hNCT and APPC100

The formation of a Nicastrin-APPC100 complex was investigated by cell-free co-expression. In vitro reactions were performed at 20 °C overnight with 0.2% Brij35. The supernatant of each reaction was incubated with Ni-NTA resin, washed with 30×CV of washing buffer (containing 20 mM Imidazole and 2×CMC Brij35), and eluted using MCAC300 Buffer (containing 300 mM Imidazole and 2×CMC Brij35). Samples were normalized for volume.

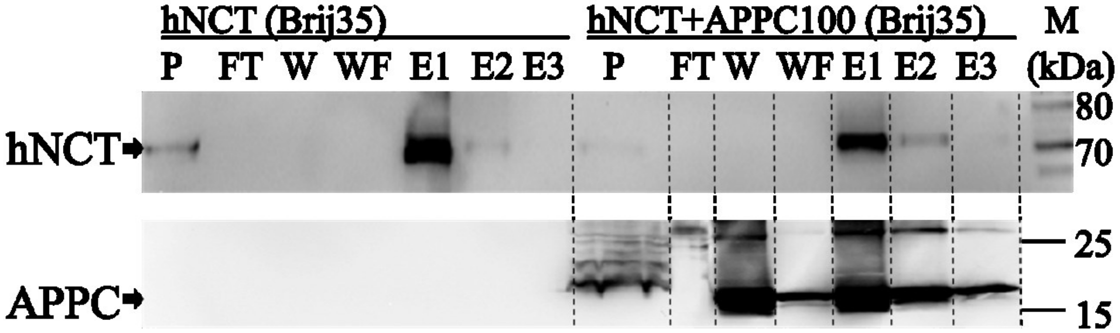

Supplementary Figure S4 Cell-free expression of hNCT and co-expression of hNCT with APPC100 in Brij35.

Top: 10 % SDS-PAGE, Western blot on His-tag.
Bottom: 15% SDS-PAGE, Western blot on Flag-tag.
P: Pellet, FT: flow through, W: wash, WF: final wash, E1-E3: Imidazole elutions.

A co-elution of hNCT (Top) and APPC100 (Bottom) was observed in the elution fractions. There are three pairs of histidine residues in the Flag-tagged APPC100 sequence, which could be responsible for a residual binding of APPC100 to Ni-NTA resin.

Supplementary Information

Control for pull-down assay APPC100-Nicastrin interaction

A 60-kDa membrane protein was utilized as a reference to detect the unspecific binding introduced by hydrophobic interaction between the TMs. The pull-down assay was performed using Flag resin.

There was no observation of the co-elution of the reference protein with APPC100 indicating the APPC100 did not randomly aggregated with other membrane proteins.

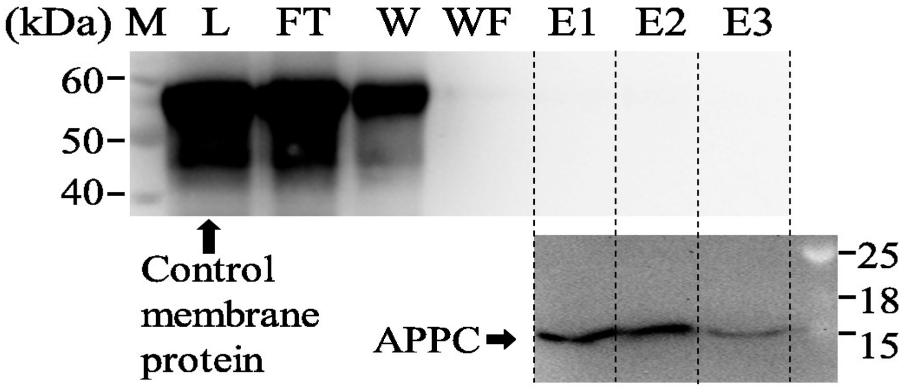

Supplementary Figure S5 Pull-down assay of control membrane protein and APPC100.

Top: 10 % SDS-PAGE, Western blot of the reference membrane protein.

Bottom: 15 % SDS-PAGE, Western blot of APPC100.

Supplementary Information

Interaction of FOS14-purified hNCT and APPC100

The binding of APPC100 to Nicastrin was analysed by Microscale Thermophoresis (MST). Interaction of the detergent containing buffer system with the labelling reagent was measured as a control (**Supplementary Fig. S6** a).

In a second binding experiment (**Supplementary Fig. S6** b) APPC100 was labelled and titrated with Nicastrin (reverse experiment). The biphasic response could not be deconvoluted effectively: By fitting the first 12 data points an apparent K_d in the range of 0.1-0.2 μM was obtained, while the second low-affinity binding event happened with apparent K_d over 1 μM.

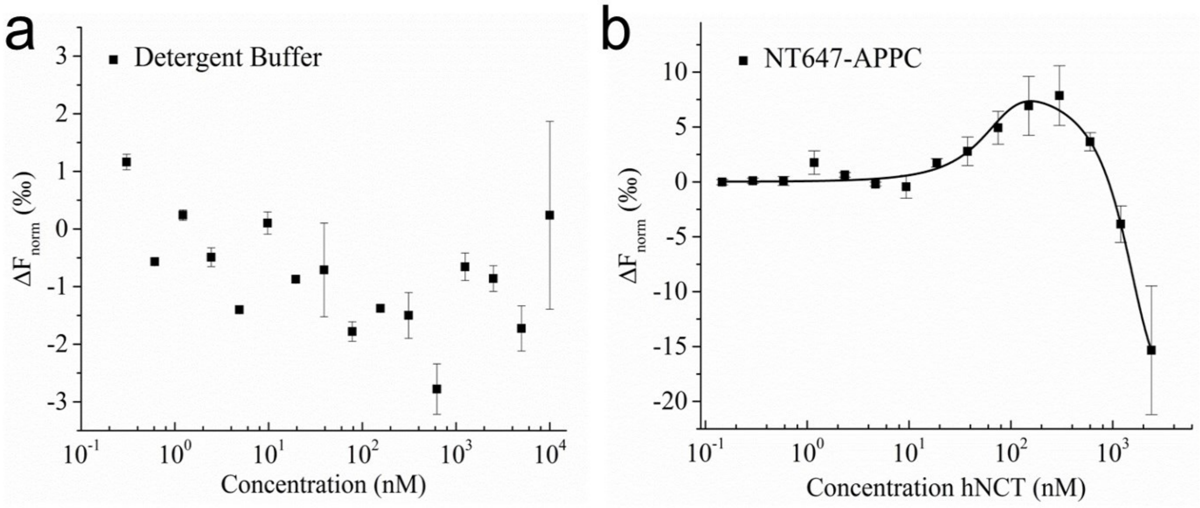

Supplementary Figure S6 MST measurements of detergent buffer and NT-647 labelled APPC100.

(a) Titration of detergent buffer (FOS14) to dye-labelled protein did not show any binding events.

(b) Titration of hNCT to a constant amount of NT647-NHS labelled APPC100.

Error bars represent the s.d. of each data point calculated from three independent thermophoresis measurements.
